# Monocytes educated by cancer-associated fibroblasts secrete exosomal miR-181a to activate AKT signaling in breast cancer cells

**DOI:** 10.1186/s12967-022-03780-2

**Published:** 2022-12-03

**Authors:** Katayoon Pakravan, Majid Mossahebi-Mohammadi, Mohammad H. Ghazimoradi, William C. Cho, Majid Sadeghizadeh, Sadegh Babashah

**Affiliations:** 1 Department of Molecular Genetics, Faculty of Biological Sciences, Tarbiat Modares University, P.O. Box: 14115-154, Tehran, Iran; 2 Department of Hematology, School of Medical Sciences, Tarbiat Modares University, Tehran, Iran; 3 Department of Clinical Oncology, Queen Elizabeth Hospital, Kowloon, Hong Kong China

**Keywords:** Breast cancer, Cancer-associated fibroblasts, Tumor-associated macrophages, Immunosuppressive tumor microenvironment, Exosomes, AKT signaling

## Abstract

**Background:**

Cancer-associated fibroblasts (CAFs), one of the major components of the tumor stroma, contribute to an immunosuppressive tumor microenvironment (TME) through the induction and functional polarization of protumoral macrophages. We have herein investigated the contribution of CAFs to monocyte recruitment and macrophage polarization. We also sought to identify a possible paracrine mechanism by which CAF-educated monocytes affect breast cancer (BC) cell progression.

**Methods:**

Monocytes were educated by primary CAFs and normal fibroblast (NF); the phenotypic alterations of CAF- or NF-educated monocytes were measured by flow cytometry. Exosomes isolated from the cultured conditioned media of the educated monocytes were characterized. An in vivo experiment using a subcutaneous transplantation tumor model in athymic nude mice was conducted to uncover the effect of exosomes derived from CAF- or NF-educated monocytes on breast tumor growth. Gain- and loss-of-function experiments were performed to explore the role of miR-181a in BC progression with the involvement of the AKT signaling pathway. Western blotting, enzyme-linked immunosorbent assay, RT-qPCR, flow cytometry staining, migration assay, immunohistochemical staining, and bioinformatics analysis were performed to reveal the underlying mechanisms.

**Results:**

We illustrated that primary CAFs recruited monocytes and established pro-tumoral M2 macrophages. CAF may also differentiate human monocyte THP-1 cells into anti-inflammatory M2 macrophages. Besides, we revealed that CAFs increased reactive oxygen species (ROS) generation in THP-1 monocytes, as differentiating into M2 macrophages requires a level of ROS for proper polarization. Importantly, T-cell proliferation was suppressed by CAF-educated monocytes and their exosomes, resulting in an immunosuppressive TME. Interestingly, CAF-activated, polarized monocytes lost their tumoricidal abilities, and their derived exosomes promoted BC cell proliferation and migration. In turn, CAF-educated monocyte exosomes exhibited a significant promoting effect on BC tumorigenicity in vivo. Of clinical significance, we observed that up-regulation of circulating miR-181a in BC was positively correlated with tumor aggressiveness and found a high level of this miRNA in CAF-educated monocytes and their exosomes. We further clarified that the pro-oncogenic effect of CAF-educated monocytes may depend in part on the exosomal transfer of miR-181a through modulating the PTEN/Akt signaling axis in BC cells.

**Conclusions:**

Our findings established a connection between tumor stromal communication and tumor progression and demonstrated an inductive function for CAF-educated monocytes in BC cell progression. We also proposed a supporting model in which exosomal transfer of miR-181a from CAF-educated monocytes activates AKT signaling by regulating PTEN in BC cells.

**Supplementary Information:**

The online version contains supplementary material available at 10.1186/s12967-022-03780-2.

## Significance

- Cancer-associated fibroblasts (CAFs), as a major component of tumor stroma, affect tumor cell behavior in different manners. Even though it has been shown that CAFs support tumor growth, not much is known about how they contribute to immunosuppression in the tumor microenvironment (TME) of breast cancer (BC).
- Tumor stromal cells may secrete paracrine factors, which can affect the distinct layers of the neoplastic population, resulting in phenotypic and signaling pathway alterations throughout the tumor cohort.
- CAFs take a crucial part in shaping the tumor immunosuppressive microenvironment in BC by recruiting monocytes and affecting their polarization states.
- Our findings propose that exosomal transfer of miR-18a1 may be a novel mechanism underlying the paracrine effects of tumor-educated monocytes and may provide a way for exosomes to promote tumor progression within the microenvironment of BC cells.

**Supplementary Information:**

The online version contains supplementary material available at 10.1186/s12967-022-03780-2.

## Background

The tumor microenvironment (TME) is a complex and continuously evolving entity composed of stromal cells, immune cells, and blood vessels arranged in the extracellular matrix. The reciprocal and dynamic crosstalk between the TME components and tumor cells contributes to tumorigenesis [1]. Cancer-associated fibroblasts (CAFs), which are a prominent part of the tumor stroma, not only provide physical support for tumor cells to promote tumor growth and progression, but they also contribute to an immunosuppressive TME by affecting many immune cells [2, 3]. Tumor-associated macrophages (TAMs), which originate from circulating monocyte precursors, are the most abundant immune cell type in close proximity to the CAF-populated areas, indicating a close association between these two major cell populations in the stroma of tumors, where they often exert protumorigenic functions [3, 4].

Cancer locally educates TAMs to distinguish them from monocytes and tissue-resident macrophages [5]. The mutual interactions with tumor cells and the stromal microenvironment contribute to the phenotypic polarization of TAMs. The M1/M2 polarization of macrophages is endowed with a repertoire of tumor-promoting capabilities involving tumor growth and metastasis, tissue remodeling, and immunosuppression [6]. Breast cancer (BC) is characterized by having a large population of TAMs, most of which exhibit the M2 phenotype. Both CAFs and TAMs support tumor progression and an increased number of either is strongly associated with poor clinical outcomes [7]. CAFs and TAMs do more than just reciprocal communication with the tumor cells; they also interact with each other in a dynamic way in the tumor *milieu* [4]. Numerous studies have indicated that CAFs play a crucial role in monocyte recruitment and M2 polarization in different types of tumors [8–11], such as BC [12]. Investigating TME-induced macrophage polarization and communication between TAMs and tumor cells is crucial for further understanding of TAM-related pro-tumor outcomes and the potential development of novel therapeutic strategies [13].

Exosomes, a subclass of membrane-derived extracellular vesicles with a size range of 30–150 nm in diameter, are produced and released by all types of cells into the extracellular *milieu* [14]. Exosome-mediated transfer of functional coding and non-coding RNAs is a mechanism of genetic exchange between cells in the TME, thereby affecting tumor development and progression [15–18]. However, the function of TAM-derived exosomes in the BC-immunosuppressive microenvironment remains to be clarified. MicroRNAs (miRNAs, miRs) are a class of non-coding, endogenous, small RNAs that negatively regulate gene expression by inducing degradation or translational repression of target mRNAs [19]. From a therapeutic intervention perspective, intercellular communications mediated by exosomal miRNAs are attracting increasing attention due to their contributions to tumor progression by reprogramming the TME [20].

There is little known about the functional effects of the exosome-mediated transfer of miRNAs released from tumoral monocytes on BC pathogenesis. In this study, we first elucidated that CAFs obtained from invasive BC recruited monocytes and induced an M2-like pro-tumoral phenotype, promoting BC progression. Next, we aimed to shed light on the mechanism by which the exosome-mediated transfer of miR-181a secreted by CAF-educated monocytes activates AKT signaling in BC cells.

## Materials and methods

### Clinical samples and processing

The peripheral blood samples from 40 invasive breast ductal carcinoma patients who had not received any chemotherapeutic treatment before surgery were collected in the study. A cohort of 35 age-matched healthy control women with no evidence of any personal or family history of BC participated in this study. A record of the clinicopathological parameters of BC patients is summarized in Additional file 1: Table S1. To harvest the plasma samples, around 5 mL of blood samples from each participant were centrifuged at 3000×*g* for 10 min at 4 °C, and then stored at − 80 °C until use. To isolate the stromal fibroblasts, primary cancer tissues were obtained from three female BC patients with histological grade III invasive ductal carcinoma who had undergone mastectomy. Normal breast tissues were obtained from three healthy women undergoing reduction mammoplasty. This study was approved by the Ethics Committee of Tarbiat Modares University and written informed consent was obtained from the participants.

### Isolation, characterization, and culture of primary fibroblasts

Fibroblasts were isolated enzymatically from both normal and cancerous breast tissues using collagenase A as previously described [21] and were maintained in Dulbecco’s Modified Eagle’s medium nutrient mixture F12 (DMEM/F12) supplemented with 10% fetal bovine serum (FBS). Immunophenotyping of patient-derived CAFs was performed for positive expression of α-smooth muscle actin (α-SMA), fibroblast-activation protein (FAP), and negative expression of CD31 to exclude endothelial cell contamination using flow cytometry (BD Biosciences). To prepare the conditioned media (CM) of cancerous and normal cultured fibroblasts (CAF- and NF-CM, respectively) for monocyte treatments, stromal fibroblasts derived from tissue specimens were passaged 4–6 times. The expressions of CAF markers (α-SMA and FAP) and CAF-derived cytokines interleukin (IL)-6 and transforming growth factor (TGF)-β as were also measured by western blotting over the course of passaging the cells. When fibroblasts reached a confluency of > 80%, the cells were serum starved. After 48 h, the CM were collected, pooled, and centrifuged at 300×*g* for 10 min and then further centrifuged at 10,000×*g* for 30 min to eliminate residual cells and cellular debris, respectively.

### Monocyte isolation and characterization

Low-density mononuclear cells were first separated from the peripheral blood of healthy volunteers using a Ficoll-Hypaque density gradient. CD14^+^ monocytes were isolated using a magnetic bead-based positive selection system (Miltenyi Biotech, Germany) with a purity of > 90%, as confirmed by flow cytometric analysis. CD14^+^ monocytes were cultured in Roswell Park Memorial Institute (RPMI)-1640 medium supplemented with 10% FBS alone as control monocytes or were educated with CM derived from CAFs or NFs (CM:RPMI, 1:1) for 7 days. Cultured monocytes were stained with antibodies against CD163, CD206, PD1, CD14, as well as HLA-DR and then analyzed by flow cytometry (BD Biosciences).

### T-cell isolation and expansion

Peripheral blood mononuclear cells (PBMCs) were isolated from healthy individuals’ peripheral blood by Ficoll-Hypaque density gradient separation. The isolated PBMCs were then cultured in RPMI-1640 medium containing 10% FBS at 37 °C for 2 h. Subsequently, the adherent cells were removed and T-cells were isolated by nylon wool columns. T-cells were stimulated with 5 μg/mL of phytohemagglutinin (PHA) and expanded in RPMI-1640 medium supplemented with 10% FBS for 7–10 days.

### Cell cultures

The human BC cell lines MDA-MB-231 and MCF-7 were cultivated in DMEM supplemented with 10% exosome-depleted FBS. The human monocyte cell line THP-1 was cultured in RPMI medium containing 10% FBS. All cells were cultured with 100 U/mL penicillin and 100 μg/mL streptomycin at 37 °C in a 5% CO_2_ humidified atmosphere. The monocytic THP-1 cells were differentiated into macrophages by 24 h incubation with 150 nM phorbol 12-myristate 13-acetate (PMA, Sigma-Aldrich) in RPMI medium. Macrophage M2 polarization was obtained by incubation of THP-1 M0 macrophages with 20 ng/mL of interleukin (IL)-4 for 48 h.

### Isolation and characterization of exosomes

Exosomes were isolated from the supernatant of monocytes educated with the CM derived from CAFs or NFs by differential centrifugation as we previously described [17]. Briefly, monocytes were maintained in bovine serum albumin (BSA) or serum-free medium 48 h before supernatant collection. The cell culture supernatants were collected, and centrifuged at 300×*g* for 10 min to eliminate residual cells and at 10,000×*g* for 30 min to further remove cells and debris. The supernatant was filtered through a 0.22-μm filter to remove any vesicles larger than 200 nm. The filtered supernatant was subjected to ultracentrifugation at 100,000×*g* for 70 min at 4 °C. To further eliminate contaminating protein, the exosome pellet was re-suspended in PBS and centrifuged again at 100,000×*g* for 70 min at 4 °C. Finally, the exosome-enriched pellets were re-suspended in PBS and stored at − 80 °C until use.

The quantity of exosomes was expressed as exosome-associated proteins using the BCA method. Exosome-specific surface markers CD9 and CD81 were detected by western blotting as we previously described [17]. The morphology of exosomes was observed using transmission electron microscopy (TEM, LEO 906 Zeiss 100 kV, Germany). The size distribution of the purified exosomes was also determined by dynamic light scattering (DLS) using a Zetasizer Nano ZS (Malvern Instrument, UK).

### Cellular uptake of purified exosomes

Fluorescent labeling of purified exosomes was performed using a PKH26 Red Fluorescent Cell Linker Kit (Sigma-Aldrich) according to the manufacturer’s instructions with some modifications. The labeled exosomes were added to a subconfluent layer of BC cells and incubated at 37 °C for 6 h. Then, the cells were washed twice with PBS and fixed with 4% paraformaldehyde. For nuclear staining, DAPI (4′, 6-diamidino-2-phenylindole, Sigma-Aldrich) was used. The uptake of exosomes was imaged using a spectral confocal microscope (Nikon Eclipse TiE).

### CFSE proliferation assay

To assess the effects of CAF- or NF-educated monocytes on T lymphocyte proliferation, T-cells were labeled with carboxyfluorescein succinimidyl ester (CFSE) and were co-cultured with either control monocytes or with monocytes educated with CAFs or NFs. In parallel, T-cells were incubated with exosomes derived from NFs or CAFs. After 72 h, the proliferation of CFSE-labeled T cells was evaluated by flow cytometry and compared to the control T-cells.

### Transient transfection

BC cells were transiently transfected with 25 nM miR-181a mimic or negative controls (NC); miR-181a inhibitor or scramble (Sc) using Lipofectamine^®^ 2000 (Invitrogen, USA). To perform a luciferase reporter assay, BC were transfected with 2 µg of the psi-CHECK2 luciferase reporter plasmid (Promega). Then, luciferase-expressing BC cells were co-cultured with monocytes and were maintained in the control medium, NF-CM, or CAF-CM. After 24 h, cell lysate was collected and added into a 96-well plate. The luciferase activity was measured using the Luciferase Reporter Assay System (Promega Corp., Madison, WI, USA).

### Cell cycle analysis

BC cells were transfected with miR-181a mimic, miR-181a inhibitor, or the corresponding negative controls as described above. Additionally, another group of BC cells was treated with 100 µg/mL exosomes derived from CAFs or NFs. After 36 h, BC cells were harvested, treated with Triton X100 and RNase A, and then stained with propidium iodide (PI, Sigma). Afterward, cell cycle distribution was analyzed via a flow cytometer (BD Biosciences).

### Cell migration assays

A confluent monolayer of serum-starved BC cells plated into a 12-well plate was subjected to a single-scratch wound by using a sterile pipette tip. The cells were incubated with 100 µg/mL exosomes derived from control monocytes, NEMo, or TEMo and compared to PBS-treated control cells. The cell migration distance was measured and imaged. A cell transwell assay was also performed using 24-well transwells (Corning, USA). The corresponding treated or transfected cells were seeded into the upper chambers in 100 µL of FBS-free medium. As a chemoattractant, the medium with 10% FBS was added to the bottom part of the chambers. Cells were fixed and stained using 1% crystal violet dissolved in methanol 24 h after incubation. The cells that migrated through the membrane and stuck to the lower surface of the membrane were imaged and counted.

### Determination of intracellular reactive oxygen species production

The intracellular reactive oxygen species (ROS) production levels were measured by adding the 2′, 7′-dichlorofluorescein diacetate (DCFDA) (ab113851, Abcam) to the cell suspension according to the manufacturer’s protocol. The fluorescent intensity was measured by flow cytometry (BD FACS Canto II, BD Bioscience) and analyzed by FlowJo Software 7.6.2.

### Enzyme-linked immunosorbent assays

Quantitative measurements of secreted IL-10 and IL-12 cytokines were performed on the culture supernatants of THP-1 M0 macrophages, M2 macrophages (serve as positive control), and NF- or CAF-educated M2 macrophages for 48 h, using enzyme-linked immunosorbent assay (ELISA). All cell culture supernatants were used undiluted.

### In silico analysis for prediction of miR-181a candidate target genes

In order to predict the potential targets of miR-181a, we used TargetScan 8.0 (http://www.targetscan.org), miRTargetLink Human (https://ccb-web.cs.uni-saarland.de/mirtargetlink) and RNAhybrid (https://bibiserv.cebitec.uni-bielefeld.de/rnahybrid) online tools. The DNA Intelligent Analysis (DIANA)-miRPath v3.0 (http://diana.imis.athena-innovation.gr/DianaTools) algorithm was used to show the miRNA regulatory roles and to find the significant Kyoto Encyclopedia of Genes and Genomes (KEGG) molecular pathways.

### RNA extraction and reverse transcription quantitative PCR

Total RNA was isolated using TRIzol reagent (Invitrogen, USA) according to the manufacturer’s recommendation and treated with RNase-free DNase (Fermentase, Lithuania). RNA was then reverse-transcribed into complementary DNA (cDNA) using the PrimeScript 1st strand cDNA synthesis kit (TAKARA, Japan). To quantify miRNA, a poly(A) tail was initially added to the extracted total RNA by using polyA polymerase enzyme (NEB), and cDNA was then synthesized by using an anchored oligo (dT) primer as described previously [22]. Reverse transcription quantitative PCR (RT-qPCR) was conducted on an ABI Step One Detection System (Applied Biosystems, USA). The relative expression was normalized to *U48* small nuclear RNA (snRNA) and *GAPDH* using the 2^−ΔCt^ and 2^−ΔΔCt^ methods [23].

### Western blotting

Total protein was extracted from the cells or exosomes using radioimmunoprecipitation (RIPA) lysis buffer. Equal amounts of proteins were separated by 12% SDS-polyacrylamide gel electrophoresis (SDS-PAGE) and transferred to polyvinylidene difluoride (PVDF) membranes. For membrane blocking, 5% skim milk was used for 1 h at room temperature. The primary antibody incubation was performed for 12 h at 4 °C and then followed by horseradish peroxidase (HRP)-conjugated secondary antibody incubation for 1 h at room temperature. The membranes were subjected to chemiluminescence using an ECL Kit (Amersham, UK). β-actin was used as a loading control.

### In vivo tumor xenograft model

All animal experimental procedures were approved by the Committee for Animal Research of the University. A total of 5 × 10^6^ MDA-MB-231 BC cells alone or mixed with 200 µg/mL exosomes (derived from TEMo or NEMo) were suspended in 100 µL serum-free DMEM and matrigel (1:1 ratio) and were subcutaneously injected into the oxter of 6-week old female BALB/C athymic nude mice. Tumor volume was monitored at every 7-day interval. The tumor volume was calculated using the formula: V (mm^3^) = (L × W^2^)/2. After 4 weeks, the mice were sacrificed to evaluate tumor growth.

### Hematoxylin and eosin staining and immunohistochemistry

Formalin-fixed tumor tissues were embedded in paraffin and cut into thin sections. The tissue sections were stained with hematoxylin and eosin (H&E) using standard procedures. For immunohistochemical (IHC) staining, tissue sections were dewaxed with xylene and then rehydrated in graded series of ethanol. Antigen retrieval was subsequently performed by microwave heating in sodium citrate buffer at pH 6.0. To block endogenous peroxidase activity, the sections were immersed in 3% H_2_O_2_ for 30 min. The sections were incubated with primary monoclonal antibodies against α-SMA (ab7817, Abcam) and Ki-67 (sc-23900, Santa Cruz Biotechnology) at 4 °C overnight. After washing, the sections were incubated with HRP-conjugated secondary antibody for 1 h, and reactive products were visualized by staining with 3, 3′-diaminobenzidine (DAB). The images were captured using an inverted fluorescence microscope (Olympus CKX41, Japan) with appropriate magnification.

### Statistical analysis

Data are expressed as the mean ± standard deviation (SD) of at least three experiments. Statistical significance was calculated by Student’s *t*-test when comparing two groups or by one-way or two-way analysis of variance (ANOVA) when comparing three or more groups. A *p*-value of < 0.05 was considered statistically significant.

## Results

### Primary CAFs recruit monocytes and induce a pro-tumoral phenotype resembling M2 macrophages

**Fig. 1:**
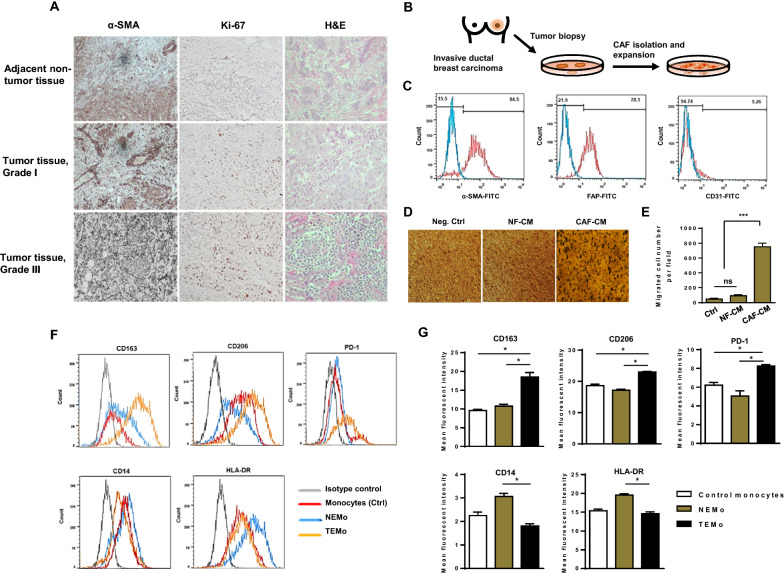
Stromal fibroblasts isolated from BC tissues exhibit characteristics of CAFs, effectively recruit monocytes and subsequently affect their polarization states. **A** Representative photomicrographs of H&E staining and IHC staining of α-SMA (a CAF-specific marker) and Ki67 (a proliferation marker) positive cells in breast tumor and adjacent non-tumor tissues (×100). Immunohistochemically, the presence of α-SMA demonstrated the presence of stromal myofibroblasts surrounding the cancer nests, which were distributed among the invasive cancer cells in the heterogeneous cancer tissue. **B** A schematic illustration of primary CAF isolation and expansion in vitro. **C** Flow cytometry staining of primary cultured CAFs with anti-α-SMA, anti-FAP, and anti-CD31. CAFs were characterized by the expression of α-SMA and FAP but lacked the expression of endothelial cell marker CD31 by flow cytometry. **D**, **E** To elucidate that monocytes are functionally recruited by CAFs, serum-starved monocytes were allowed to migrate for 12 h toward CMs from CAFs or NFs and compared to the negative control. **D** Representative photomicrographs of the migration potential of monocytes in different conditions assessed using the transwell assay. **E** Quantitative assessment of migrated cells showed that monocytes were effectively recruited by CAF-CM. Representative flow cytometry histograms (**F**) and bar graphs of mean fluorescent intensity (**G**) show that the expression levels of CD163, CD206, and PD-1 were considerably higher in TEMo than in NEMo or control monocytes, while the expression levels of HLA-DR and CD14 were significantly lower in TEMo compared to NEMo. Columns, mean of three different experiments; bars, SD. *P-value < 0.05, ***P-value < 0.001

To investigate the relationship between CAFs and the malignance phenotype, we first studied the expression of α-SMA (a myoepithelial cell marker) and Ki67 (a marker of proliferating cells) in tumor and non-tumor tissues obtained from BC patients. IHC assay performed on tissue samples confirmed that α-SMA is localized, particularly in adjacent stromal cells, which are mainly comprised of stromal myofibroblasts. Results also indicated that the expression of α-SMA has a significant association with histopathological grade (Fig. 1A). As CAFs appear to be major tumor microenvironment components facilitating tumor progression, we aimed to clarify the function of CAFs isolated from patients who have been diagnosed with advanced grade tumors. To this end, CAFs were isolated from three primary BC tissues (Grade III) obtained from patients undergoing mastectomy (Fig. 1B). As healthy counterparts, we used normal fibroblasts (NFs) isolated from normal breast tissues obtained from patients undergoing reduction mammoplasty. Primary cultures of both of those isolated fibroblasts were established. The evaluation of cell surface markers by flow cytometry indicated the positive expression of α-SMA and FAP but the lack of expression of vessel marker CD31 in isolated CAFs (Fig. 1C). Additionally, western blot analysis revealed the expression of CAF-specific markers (α-SMA and FAP) and CAF-derived cytokines (IL-6, and TGF-β) over the course of passaging, confirming CAF activation status throughout the experiments (Additional file 1: Fig. S1).

Stromal cells have been demonstrated to affect monocyte recruitment and polarization in addition to altering their functions in the TME [24]. To evaluate whether CAFs recruit monocytes, we isolated monocytes from the peripheral blood of healthy individuals and performed transwell migration assays. The bottom chambers contained conditioned media (CM) collected from CAFs and NFs, thus serving as chemoattractants. We observed that monocytes migrate toward CAF-CM in the bottom chambers, suggesting that CAFs may effectively recruit monocytes (Fig. 1D, E). Additionally, to explore whether CAFs were able to induce a pro-tumoral phenotype in monocytes, CD14^+^ cells isolated from healthy individuals were subjected to differentiation following treatment with CAF-CM. Hereafter, monocytes educated by CM derived from tumoral CAFs are denoted as tumor-educated monocytes or TEMo, and monocytes co-cultured with CM derived from NFs are referred to as normal fibroblast-educated monocytes or NEMo. After 7 days of culture, the expression M2 macrophage markers CD163 and CD206 were higher in TEMo than in NEMo. In addition, the expression of programmed cell death protein 1 (PD-1) was higher in TEMo than in NEMo. In fact, NF-educated cells’ expressions of PD-1, CD163, and CD206 were similar to control monocytes. On the other hand, the expression of CD14 was much higher in NEMo than in TEMo. In addition, the major histocompatibility complex (MHC) class II (HLA-DR) expression of TEMo is much lower than that of NF-educated cells (*P* < 0.05, Fig. 1F, G). All of these results suggest that CAFs may recruit monocytes and affect their polarization states.

### CAFs promote THP-1 polarization into anti-inflammatory M2 macrophages

**Fig. 2:**
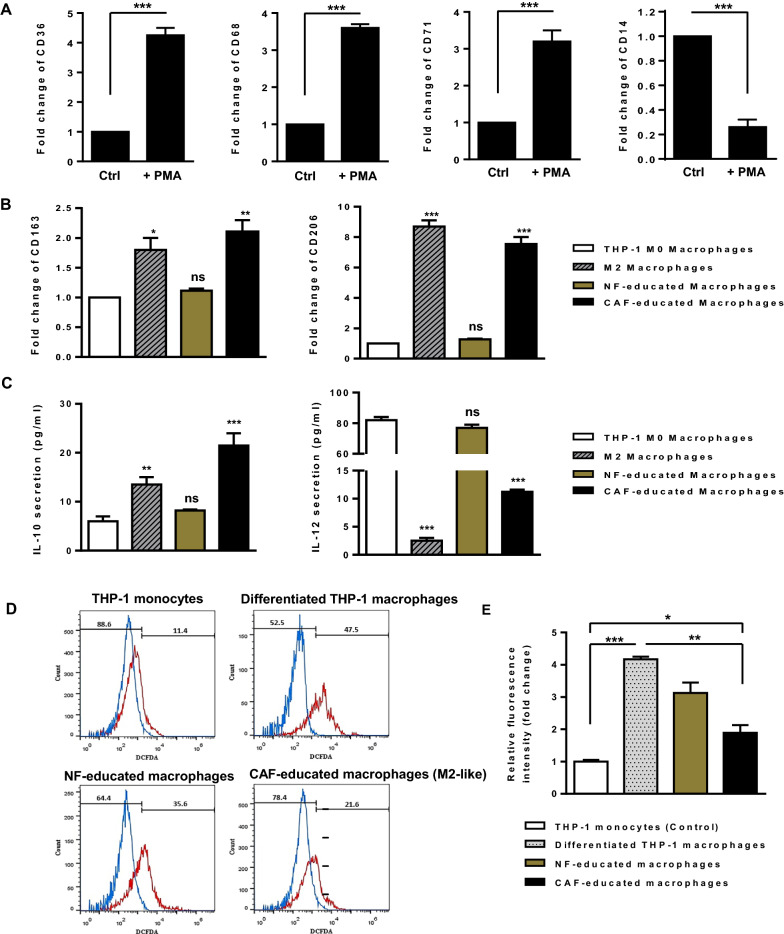
CAFs induce the polarization of THP-1 cells into anti-inflammatory M2-like macrophages. **A** After 24 h of incubation with 150 nM PMA, the monocyte-to-macrophage differentiation was confirmed by the elevated transcript levels of recognized macrophage markers CD36, CD68, and CD71. The decreased transcript level of CD14 in PMA-treated THP-1 cells further confirmed that the THP-1 monocytes were differentiated into macrophage-like cells. **B** Transcript expression levels of M2 macrophage markers CD163 and CD206 in THP-1 macrophages incubated with CAF-CM were significantly increased compared with those in THP-1 M0 macrophages alone or incubated with NF-CM after 48 h. As a positive control, THP-1 macrophages were stimulated with 20 ng/mL of IL-4 for 48 h. **C** Protein secretion levels of the anti-inflammatory cytokine IL-10 and the pro-inflammatory cytokine IL-12 were assessed by ELISA. THP-1 macrophages incubated with CAF-CM as well as positive control M2 macrophages were identified by IL-10^high^ IL-12^low^ phenotype when compared to THP-1 M0 macrophages alone or incubated with NF-CM, confirming the potential of CAFs in promoting the polarization of THP-1 cells into anti-inflammatory M2-like macrophages after 48 h. **D**, **E** The relative levels of intracellular ROS production during macrophage polarization. The amount of ROS produced by differentiated THP-1 macrophages increased noticeably when monocytes were treated with PMA. Importantly, ROS generation in CAF-educated macrophages was lower than that in macrophages educated by NF-CM after 48 h. Even though the level of ROS diminishes during M1/M2 polarization, CAF-induced M2-like macrophages were found to produce higher levels of ROS than THP-1 control monocytes. Representative flow cytometry histograms (**D**) and bar graphs of relative DCFDA fluorescence intensity (**E**) imply that CAFs contribute to M2 polarization in part by increasing ROS production, as M2 macrophages require a level of ROS for proper polarization. Columns, mean of three different experiments; bars, SD. ns: non-significant, *P-value < 0.05, **P-value < 0.01, ***P-value < 0.001

To explore the capability of CAF to induce M2 polarization of macrophages, the human monocyte cell line THP-1 was differentiated into macrophages by incubation in the presence of 150 nM PMA. After 24 h, cells became adherent and displayed an irregular cell shape characteristic of cells undergoing differentiation. As shown in Fig. 2A, qRT-PCR revealed that the expression levels of recognized macrophage markers CD36, CD68, and CD71 were found to be higher as the macrophages differentiated (*P* < 0.001). The expression of CD14 was also decreased (*P* < 0.001), further confirming the monocyte-to-macrophage differentiation (Fig. 2A). Afterward, THP-1 M0 macrophages were incubated with either CAF- or NF-CM and compared to positive control cells (i.e., THP-1 M0 macrophages stimulated with 20 ng/mL of IL-4 for 48 h). The M2 polarization of macrophages was assessed by studying the transcript and protein levels of several M2 macrophage markers. After 48 h of incubation, the transcript levels of CD163 (*P* < 0.01) and CD206 (*P* < 0.001) were found to be significantly increased in CAF-CM-incubated THP-1 macrophages (Fig. 2B). Consistently, protein secretion level of anti-inflammatory cytokine IL-10 was increased in culture supernatants of CAF-educated macrophages (*P* < 0.001), whereas protein secretion level of pro-inflammatory cytokine IL-12 was found to be decreased in macrophages incubated with CAF-CM (*P* < 0.001) (Fig. 2C). These findings shed light on the potential of CAFs in promoting THP-1 polarization into anti-inflammatory M2 macrophages.

Additionally, as it was demonstrated that oxidative stress plays a role in modulating macrophage phenotype, we aimed to determine ROS status during macrophage polarization. Data showed that when monocytes were incubated with PMA, ROS production in differentiated THP-1 macrophages was markedly elevated (*P* < 0.001; Fig. 2D, E). Importantly, following CAF-CM incubation, ROS production was abolished in M2-like macrophages (P < 0.01; Fig. 2D, E). Even though ROS levels dropped during M1/M2 macrophage polarization, CAF-induced M2-like macrophages still seem to produce more ROS than THP-1 control monocytes (*P* < 0.05; Fig. 2D, E). Overall, these findings suggest that CAFs contribute to M2 polarization in part by increasing ROS production in monocytes, as M2 macrophages require a level of ROS for proper polarization.

### Isolation, characterization, and cellular uptake of exosomes derived from monocytes

**Fig. 3:**
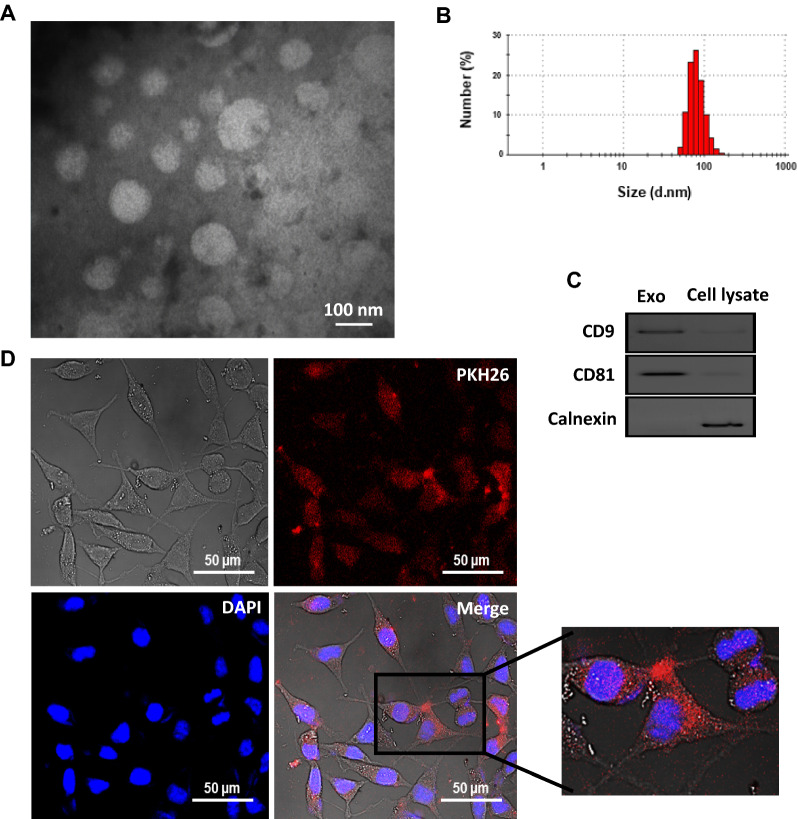
Characterization and cellular uptake of purified exosomes derived from monocytes. The morphology and size of exosomes were observed using (**A**) transmission electron microscopy. **B** Representative dynamic light scattering (DLS) number distribution measurement of purified exosomes showed a single peak at ~ 90 nm. **C** Exosome-specific surface markers CD9 and CD81 were detected in exosomes by western blotting. The cytoplasmic protein marker Calnexin was expressed in the whole cell lysate but was undetectable in the isolated exosomes, indicating that the exosome preparations were not contaminated with other vesicles such as endoplasmic reticulum ones. **D** Cellular internalization of PKH26-labeled exosomes by MDA-MB-231 BC cells was visualized and imaged under a confocal microscope. The red fluorescence in the cytoplasm showed that exosomes were uptaken by MDA-MB-231 BC cells. The nuclear staining was performed by DAPI

To uncover the paracrine effect of exosomes derived from CAF-educated monocytes on BC cells, we first isolated and purified exosomes from the conditioned media of monocytes educated with CAF or NF. The morphologically round and spherical shapes of purified exosomes with an approximate diameter ranging from ~ 50–150 nm were observed by TEM (Fig. 3A). Also, the particle size distribution of exosomes by DLS revealed a single bell-shaped size distribution with a peak at ~ 90 nm (Fig. 3B). Additionally, western blotting demonstrated that the exosome-specific tetraspanin markers CD9 and CD81 were enriched in the exosome preparation but not in the monocyte lysate. Conversely, the negative maker of Calnexin (the endoplasmic reticulum protein marker) was almost undetectable in the isolated exosomes, but highly expressed in cells (Fig. 3C). To examine whether the monocyte-derived exosomes could be taken up by BC cells, PKH26-labeled exosomes were incubated with sub-confluent MDA-MB-231 BC cells for 18 h. Confocal microscopy imaging showed that monocyte exosomes can be incorporated and internalized into the cytoplasm of MDA-MB-231 BC cells (Fig. 3D).

### CAF-educated monocytes and their derived exosomes exert immunosuppressive effects by inhibiting T-cell proliferation

**Fig. 4:**
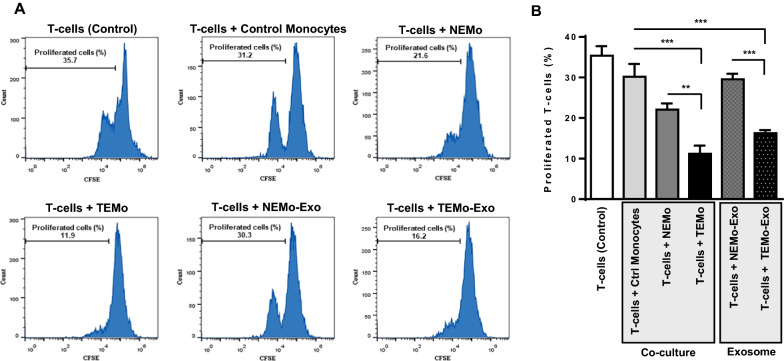
CAF-educated monocytes and their derived exosomes suppress T-cell proliferation. TEMo, NEMo, their corresponding exosomes as well as control monocytes were co-cultured with autologous peripheral T-cells which were labeled with CFSE for 72 h. Representative flow cytometry histograms (**A**) and bar graphs of proliferated T-cells (**B**) illustrated that TEMo and their derived exosomes (100 µg/mL) had a greater inhibitory effect on T-cell proliferation when compared with control monocytes, NEMo, or derived exosomes. Effector cells (i.e., monocytes) were co-cultured with target cells (labeled T-cells) at the Effector: target (E:T) ratio of 1:4. Columns, mean of three different experiments; bars, SD. **P-value < 0.01, ***P-value < 0.001

CAFs are found to be a major cause of immunosuppression, partly through disrupting T-cell function in the tumor microenvironment. First, in order to explore the functional role of CAF-educated monocytes (TEMo) in promoting immunosuppression, human autologous peripheral T lymphocytes were labeled with carboxyfluorescein succinimidyl ester (CFSE) and co-cultured with control monocytes, NEMo, or TEMo. After 72 h, flow cytometry data showed that T-cell proliferation was inhibited by TEMo more markedly than control (*P* < 0.01) and NEMo (*P* < 0.001; Fig. 4). Importantly, we found that 100 µg/mL exosomes derived from TEMO (TEMo-Exo) had a similar inhibitory effect on the proliferation of T-cells compared to that of cells co-cultured with control monocytes or treated with exosomes derived from NEMo (NEMo-Exo) (*P* < 0.01; Fig. 4). These data suggest that CAFs could exert their immunosuppressive effects on T-cells, at least in part and indirectly, through educating monocytes.

### CAF-activated monocytes lose their anti-tumoral functions and exhibit an M2 pro-tumoral phenotype, promoting BC cell proliferation

**Fig. 5:**
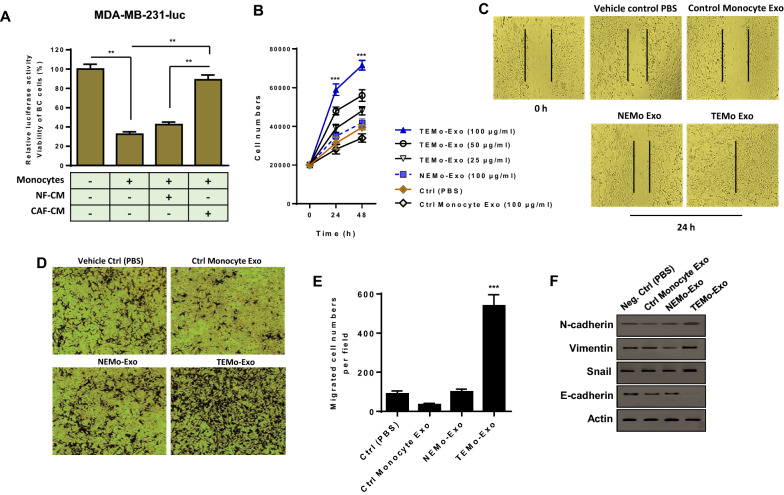
CAF-activated, polarized monocytes lose their tumoricidal properties and their derived exosomes enhance the proliferation and migration of BC cells and up-regulate the expression of EMT markers. **A** The cytotoxic activity of monocytes alone or incubated with either CAF- or NF-CM was evaluated by measuring the luciferase activity of MDA-MB-231 BC cells expressing luciferase (MDA-MB-231-luc) in a co-culture condition. Bar graphs represent the relative luciferase activity, indicative of BC viability. The reduced luciferase activity of BC cells co-cultured with control monocytes pointed to the effective anti-tumoral functions of monocytes. In contrast, when monocytes were treated with CAF-CM, these cells lost their tumoricidal properties and instead promoted BC proliferation. Columns, mean of three different experiments; bars, SD. **P-value < 0.01. **B** MDA-MB-231 BC cells were incubated with exosomes derived from all differentiated monocytes. Results showed that different concentrations of TEMo-Exo (25, 50, and 100 μg/mL) have promoting effects on the proliferation rate of MDA-MB-231 BC cells in a dose- and time-dependent manner. In contrast, BC cell proliferation was not substantially affected by incubation with 100 µg/mL NEMo-Exo as compared to PBS-treated BC cells. As expected, BC cells displayed the lowest rate of proliferation when incubated with 100 µg/mL control monocyte exosomes at the indicated time points. Points, mean of three different experiments, bars, SD. ***P-value < 0.001. **C** Representative photomicrographs of MDA-MB-231 BC cells cultured with 100 μg/mL exosomes derived from TEMo, NEMo, or control monocytes as well as in standard medium containing PBS at 24 h after scratch wounding. BC cells incubated with 100 μg/mL TEMo-Exo exhibited a considerably higher migration potential compared with those incubated with exosomes derived from control monocytes or NEMo. **D**, **E** Enhanced migration of MDA-MB-231 BC cells incubated with 100 μg/mL of TEMo-Exo, compared with the corresponding control cells. **D** Representative photomicrographs of the migration potential of MDA-MB-231 BC cells in different conditions after 24 h of incubation, assessed using the transwell assay. **E** Quantitative assessment of migrated cells showed that MDA-MB-231 BC cells treated with 100 μg/mL TEMo-Exo exhibited a significantly higher migration potential compared with cells incubated with exosomes derived from control monocytes or NEMo as well as cells cultured in standard medium containing PBS. Columns, mean of three different experiments; bars, SD. ***P-value < 0.001. **F** Western blot analysis showed up-regulation of EMT protein markers (N-cadherin, Vimentin, and Snail) and down-regulation of epithelial marker E-cadherin in MDA-MB-231 BC cells, 48 h after treatment with 100 μg/mL TEMo-Exo compared with the corresponding control cells. Actin was used as an endogenous loading control. Western blot images are representative of at least three independent experiments

M2 macrophages are typically pro-tumoral and lack the tumoricidal properties of M1 macrophages [25]. Because CAF-CM was able to induce the up-regulation of M2-specific markers, suggesting a conversion of naive monocytes into M2 macrophages (Fig. 1F, G), we first sought to investigate whether CAF-induced polarization of M2 macrophages is concomitant with the loss of tumoricidal properties of monocytes. In order to assess this phenotype, we co-cultured luciferase-expressing MDA-MB-231 BC cells (MDA-MB-231-luc) with monocytes maintained in the control medium, NF-CM, or CAF-CM. As shown in Fig. 5A, in co-cultured conditions with the control medium, the luciferase activity was remarkably diminished, suggesting the effective tumoricidal properties of monocytes. On the contrary, when monocytes were incubated with CAF-CM, these cells lost their ability to kill BC cells and instead enhanced the proliferation of BC cells. These data propose that CAFs may be able to influence the phenotype of monocytes by polarizing them to an M2 pro-tumoral phenotype, promoting BC cell proliferation.

### Exosomes secreted from CAF-educated monocytes enhance the proliferation and migration of BC cells and increase the expression of EMT markers

To investigate the effects of TEMo-Exo on BC cell proliferation and migration in vitro, MDA-MB-231 BC cells were incubated with exosomes derived from monocytes educated by CAF or NF. As shown in Fig. 5B, treatment of different concentrations of TEMo-Exo markedly resulted in a significant increase in the proliferation rate of BC cells in a dose- and time-dependent manner. On the contrary, the proliferation rate of BC cells was not significantly affected following incubation with 100 µg/mL exosomes derived from monocytes educated by NF, compared to the cells treated with vehicle control PBS. As expected, BC cells incubated with 100 µg/mL control monocyte exosomes had the lowest rate of proliferation. Using a wound healing assay, we also found that the migration of BC cells into the scratched areas of monolayers was reduced in the presence of exosomes derived from control monocytes compared to PBS-treated BC cells. Importantly, we observed that when incubated with 100 µg/mL TEMo-Exo, BC cells migrated significantly faster than BC cells incubated with NEMo-Exo or vehicle control PBS (Fig. 5C). Consistent with this observation, we found through a transwell migration assay that incubation of BC cells with 100 μg/mL TEMo-Exo led to significantly higher rates of migration through transwell membranes than those incubated with exosomes derived from control monocytes or NF-educated monocytes (Fig. 5D, E). Additionally, we showed that exposure to TEMo-Exo induced the protein expression of epithelial-to-mesenchymal transition (EMT) markers in MDA-MB-231 BC cells. As shown in Fig. 5F, the expressions of EMT markers including N-cadherin, Vimentin, and Snail were found to be up-regulated and the expression of epithelial marker E-cadherin was found to be down-regulated in BC cells by the effects of TEMo-Exo. On the contrary, neither EMT markers nor E-cadherin expression were significantly changed by the effects of NEMo-Exo, compared to that of control monocyte exosomes or PBS vehicle control. Overall, it seems that CAFs educate monocytes to secrete exosomes, which up-regulate the EMT markers and impart more migratory behavior in BC cells.

### CAF-educated monocyte exosomes promote breast cancer tumorigenicity in vivo

**Fig. 6:**
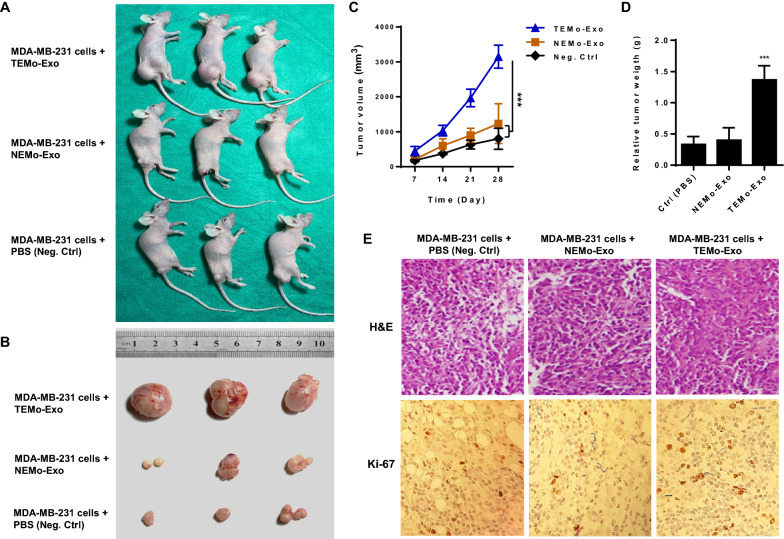
Exosomes derived from TEMo promote breast tumor growth in vivo*.* **A** Representative images of tumor-bearing nude mice subcutaneously implanted with MDA-MB-231 cells alone or mixed with exosomes derived from TEMo or NEMo. **B** Representative photographs of xenograft tumors obtained from mice at day 28 post-implantation. **C** Tumor volume at days 7, 14, 21, and 28 after subcutaneous implantation of nude mice with MDA-MB-231 cells alone or mixed with 200 µg/mL exosomes derived from TEMo or NEMo. Co-implantation of MDA-MB-231 BC cells and TEMo-Exo together resulted in a faster growth rate of tumors and a larger tumor diameter than that of mice injected with either BC cells alone or BC cells mixed with NEMo Exo. ***P-value < 0.001. **D** Tumor weight in mice implanted with MDA-MB-231 BC cells mixed with 200 µg/mL TEMo-Exo was significantly higher than in those implanted with BC cells alone or mixed with NEMo Exo at day 28 post-implantation. ***P-value < 0.001. **E** Representative photographs of H&E and IHC staining for Ki-67 on formaldehyde-fixed, paraffin-embedded MDA-MB-231-derived xenograft tumor sections from different treatment groups. IHC analysis of the cell proliferation marker Ki67 showed much higher immunoreactivity for nuclear Ki67 in the TEMo-Exo co-implantation group compared to the cell-only group or cells implanted with NEMo-Exo. **P-value < 0.01, ***P-value < 0.001

To further assess the role of TEMo-Exo in tumor growth in vivo, we established tumor models in 6-week-old BALB/c nude mice by subcutaneously injecting MDA-MB-231 cells alone or MDA-MB-231 cells mixed with 200 µg/mL exosomes derived from monocytes educated by CAF. Tumor sizes were measured three times a week, and the observations lasted over 28 days after tumor challenges. As shown in Fig. 6A–D, tumor volume and weight in mice implanted with MDA-MB-231 BC cells mixed with 200 µg/mL TEMo-Exo were significantly higher than in those implanted with BC cells alone. To test whether exosomes derived from NEMo also exert tumor-promoting effects, tumor growth in mice implanted with BC cells mixed with 200 µg/mL NEMo-Exo was measured, and the results showed that NEMo-Exo had no effect on promoting tumor growth compared to those of mice implanted with BC cells only. Furthermore, H&E staining of tumor sections revealed that cells from mice implanted with TEMo-Exo arranged more densely with a more irregular cell shape and had increased cell size when compared to the NEMo-Exo co-implantation group or negative control tumor group (Fig. 6E). As Ki-67 indicates the proliferative ability of tumors, we examined Ki-67 expression in xenograft tumor sections. Consistently, IHC results revealed that tumor tissue from mice implanted with TEMo-Exo exhibited stronger positive staining than that in the cell-only group or cells co-implanted with NEMo-Exo (Fig. 6E). Collectively, these results indicate that TEMo-Exo enhance the tumorigenicity of MDA-MB-231 BC cells in vivo, while exosomes derived from control-educated monocytes have no significant effect on the growth of xenograft tumors.

### miR-181a is up-regulated in CAF-educated monocytes and their exosomes and represents a diagnostic potential for BC patients

**Fig. 7:**
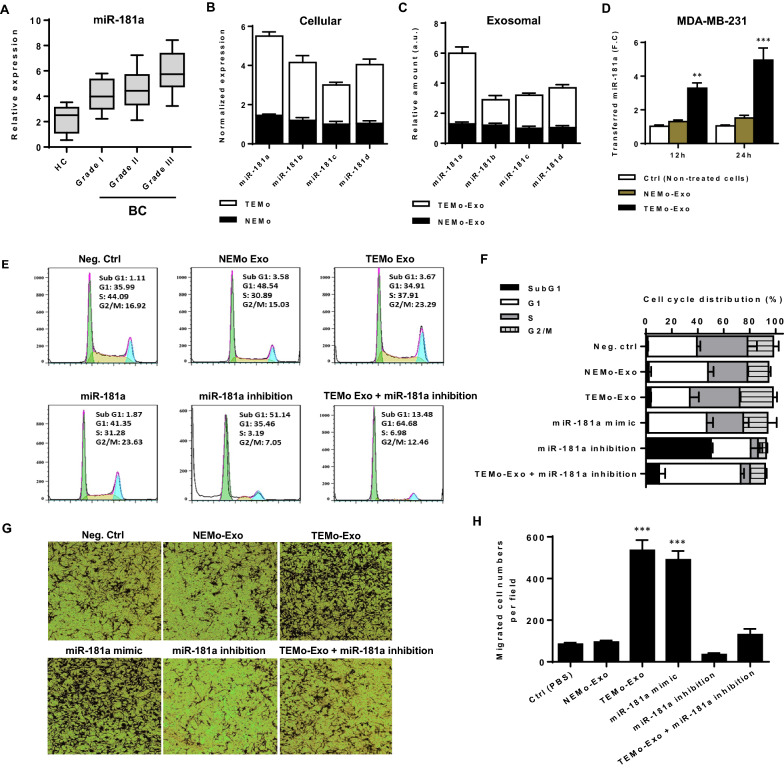
The pro-oncogenic effect of exosomes derived from TEMo on BC cell progression is partly dependent on miR-181a. **A** The relative expression level of plasma-derived circulating miR-181a in breast ductal carcinoma patients compared to healthy controls. A higher level of miR-181a was detected in BC plasma samples as compared with healthy controls, and, importantly, its expression level was correlated positively with tumor aggressiveness, as grade III tumors showed the highest expression of miR-181a with respect to grade I and II tumors. **B**, **C** Differential expression of miR-181 family members in TEMo and NEMo (**B**) as well as their corresponding exosomes (**C**). RT-qPCR results revealed a higher level of miR-181a in TEMo and TEMo-Exo compared to their normal counterparts. **D** The mean normalized ratio for miR-181a levels was assessed by RT-qPCR in MDA-MB-231 BC cells at 12 and 24 h time points. MDA-MB-231 BC cells were pre-treated with RNA polymerase inhibitor α-amanitin for 8 h before incubation with 100 μg/mL TEMo-Exo. The cells incubated with PBS and α-amanitin were used as a control. RT-qPCR results revealed that TEMo-secreted exosomal miR-181a is transferred to MDA-MB-231 BC cells in a time-dependent manner. **E**, **F** Representative flow cytometry histograms (**E**) and bar graphs (**F**) of cell cycle distribution in MDA-MB-231 BC cells in different conditions after 48 h of incubation. Flow cytometry results indicated transfection of miR-181a mimic caused BC cell progression similar to the cell cycle distribution observed in TEMo-Exo-treated BC cells. Additionally, inhibition of miR-181a resulted in the reduced distribution in the S and G2/M phases but induced an increased accumulation of cells in the sub G1 phase. Importantly, the TEMo-Exo-induced increases in S and G2/M proportions were partly prevented by reintroducing the miR-181a inhibitor, indicating that the promoting effect of TEMo-Exo on BC cell progression depends on miR-181a. **G**, **H** Representative photomicrographs (**G**) and bar graphs (**H**) illustrating the migration potential of MDA-MB-231 BC cells in different conditions after 24 h of incubation, assessed using a transwell assay. Overexpression of miR-181a recapitulated the promoting effect of TEMo-Exo on BC cell migration. However, miR-181a inhibition dramatically suppressed the phenotypes induced by TEMo-Exo in BC cells. Columns, mean of three different experiments; bars, SD. **P-value < 0.01, ***P-value < 0.001

Mounting evidence has confirmed that miRNAs play a substantial role in the initiation and progression of cancer. In this regard, the dynamic expression pattern of miRNAs may be associated with the progression of tumors [26]. To identify circulating miRNAs that may be involved in BC progression, we performed a survey of public BC data sets for miRNA expression and found that miR-181a is among miRNAs to be frequently up-regulated in BC. To explore the clinical significance of circulating miR-181a in BC, we measured the expression level of this miRNA in BC plasma samples. RT-qPCR results revealed that miR-181a expression level was significantly up-regulated in BC patients compared with healthy individuals and, importantly, miR-181a expression level was positively correlated with tumor aggressiveness (Fig. 7A). These findings confirm that plasma-derived miR-181a has the potential to be a diagnostic biomarker for BC patients.

Being enriched in exosomes, miRNAs secreted from activated monocytes in the peritumoral stroma of tumor cells may contribute to inducing the malignant behavior of tumor cells [20]. To describe the molecular mechanisms by which exosomes derived from monocytes educated with CAF promote the EMT response, we conjectured that miRNAs secreted by TEMo-Exo might account for the oncogenic effects of tumoral monocytes on BC cells. We first measured the expression of miR-181 family members in two groups of monocytes educated by CAF or NF. Fold change analysis in miRNA expression levels revealed a higher level of miR-181a in TEMo, while the expression of this miRNA did not show a significant difference compared to control-educated monocytes (Fig. 7B). We also detected a high level of miR-181a in exosomes derived from TEMo as compared to those derived from NEMo (Fig. 7C).

Since exosomes facilitate the communication between adjacent cells by transferring miRNAs [20], we sought to examine the exosomal transfer of miR-181a from educated monocytes into BC cells. To this end, MDA-MB-231 cells were incubated with 100 μg/mL exosomes derived from monocytes educated with either CAF or NF at different time points and compared to control cells. Additionally, to confirm that miR-181a is transferred from monocytes into BC cells and not transcriptionally induced, we incubated BC cells with either monocyte exosomes or PBS in the presence of α-amanitin, which is an inhibitor of transcriptional activation. As shown in Fig. 7D, a gradual increase in the level of miR-181a was observed in MDA-MB-231 cells stimulated with 100 μg/mL TEMo-Exo, while the level of this miRNA did not show a significant difference in control BC cell groups (i.e., non-treated cells or BC cells treated with NEMo-Exo). These data confirm that miR-181a is enriched in exosomes derived from monocytes educated by CAFs and is shuttled by exosomes into BC cells.

### The pro-oncogenic effects of CAF-educated monocyte exosomes depend in part on miR-181a in BC cells

To determine whether exosomes derived from TEMo promote the malignant phenotype of BC cells by transferring miR-181a, functional rescue experiments were performed. The miR-181a inhibitor was first transfected into exosome-treated BC cells. Flow cytometric analysis of the cell cycle distribution revealed that the proportion of BC cells in the S and G2/M phases in TEMo-Exo-treated BC cells was significantly higher than that in NEMo-Exo-treated control cells. Upon miR-181a silencing, the cells accumulated in the sub G1 phase, with a concomitant decrease in the proportion of cells in the S and G2/M phases. Consistently, transfection of miR-181a mimic recapitulated the promoting effects of TEMo-Exo on BC cell progression. Importantly, reintroducing the miR-181a inhibitor abolished the TEMo-Exo-caused increases in the proportions of cells in the S and G2/M phases (Fig. 7E, F). Additionally, the stimulating effect of TEMo-Exo on BC cell migration was recapitulated by miR-181a overexpression. However, the promoting effects of TEMo-Exo on the migration of BC cells were effectively weakened by exogenous inhibition of miR-181a expression (Fig. 7G, H). As miR-181a overexpression recapitulated the phenotypes induced by TEMo-Exo in BC cells, it is suggested that miR-181a shuttled by TEMo-Exo plays a vital role in promoting BC cell progression.

### miR-181a secreted from CAF-educated monocytes activates AKT signaling partly through suppressing PTEN in BC cells

**Fig. 8:**
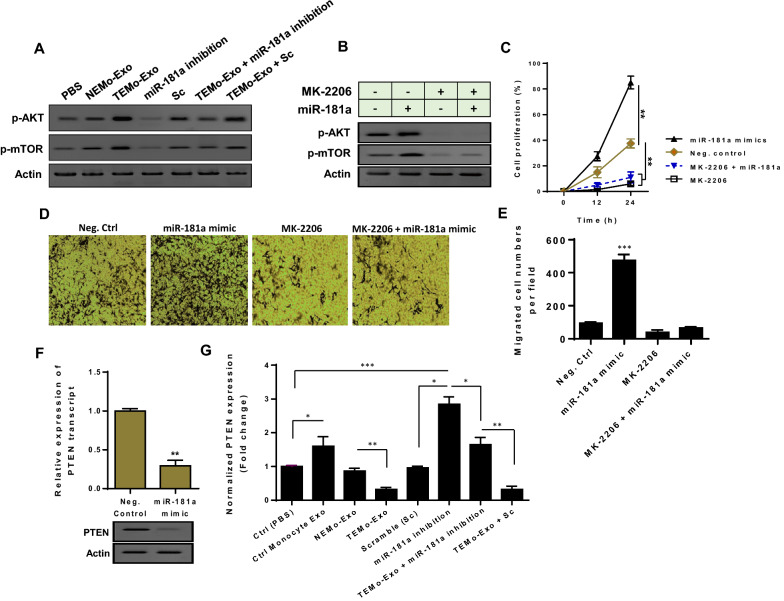
TEMo-secreted miR-181a activates AKT signaling partly by inhibiting PTEN in BC cells. **A** Western blot analysis showed up-regulation of the phosphorylated levels of AKT and mTOR in MDA-MB-231 BC cells, 48 h after treatment with 100 μg/mL TEMo-Exo compared with the corresponding control cells. Results indicated that the promoting effects of TEMo-Exo on Akt–mTOR signaling were partially abolished when the cells were transfected with the miR-181a inhibitor. **B** Western blot analysis revealed that when MDA-MB-231 BC cells were incubated with the AKT inhibitor MK-2206, the activation of the Akt–mTOR signaling caused by miR-181a was suppressed. Actin was used as an endogenous loading control. Western blot images are representative of at least three independent experiments. **C** BC cells displayed the lowest rate of proliferation when treated with MK-2206 at the indicated time points. Although transfection of miR-181a mimic considerably increased the proliferation rate of MDA-MB-231 BC cells compared to control cells, miR-181a did not increase BC cell proliferation in the cell group treated with MK-2206. Points, mean of three different experiments; bars, SD. **P-value < 0.01. **D**, **E** Representative photomicrographs (**D**) and bar graphs (**E**) illustrated that MK-2206 abrogated the promoting effect of miR-181a on BC cell migration. **F** Transfection of miR-181a mimic caused a significant reduction of PTEN expression at both mRNA and protein levels in MDA-MB-231 BC cells, suggesting that miR-181a may regulate PTEN in BC cells. **G** The mean normalized ratio for PTEN mRNA levels were measured by RT-qPCR in different conditions, 48 h after incubation. Results demonstrated that PTEN mRNA levels were considerably decreased in MDA-MB-231 BC cells incubated with 100 μg/mL TEMo-Exo compared to those cells incubated with exosomes derived from control monocytes or NEMo. In contrast, inhibition of miR-181a in MDA-MB-231 BC cells partially prevented the inhibitory effect of TEMo-Exo on PTEN expression. Columns, mean of three different experiments; bars, SD. *P-value < 0.05, **P-value < 0.01, ***P-value < 0.001

We next analyzed the intracellular signaling that might be regulated by miR-181a secreted from TEMo in BC cells. As shown in Fig. 8A, western blot analysis demonstrated a significant increase in phosphorylated AKT levels in MDA-MB-231 BC cells that were incubated with 100 μg/mL TEMo-Exo compared to control cells. To investigate the functional role of exosomal miR-181a in AKT activation, we utilized a miR-181a inhibitor and found that the promoting effect of TEMo-Exo on AKT activation was partially abrogated when MDA-MB-231 BC cells were transfected with miR-181a inhibitor. To confirm that the exosomal transfer of miR-181a functionally correlates with AKT signaling, the phosphorylated levels of mTOR were measured in BC cells. Results showed that inhibition of miR-181a reduced phosphorylated levels of mTOR in BC cells. As expected, the promoting effects of exosomes derived from TEMo on mTOR activation were partially rescued in the presence of the miR-181a inhibitor (Fig. 8A). To further investigate whether Akt signaling is critically involved in miR-181a function in BC, we inhibited the Akt activity with MK-2206, which is clinically used for the treatment of human BC [27]. Results showed that although transfection of miR-181a mimic led to the phosphorylation of Akt–mTOR, MK-2206 treatment blocked miR-181a overexpression-induced activation of Akt–mTOR signaling in BC cells (Fig. 8B). Importantly, MK-2206 blocked the effects of miR-181a on the proliferation and migration of BC cells because miR-181a overexpression did not significantly increase the proliferation and migration rates in the group of cells treated with MK-2206 (Fig. 8C–E).

Next, we explored the potential mechanism underlying miR-181a-mediated activation of Akt–mTOR signaling. Among the predicted targets of miR-181a, PTEN is a key tumor suppressor gene, dysregulation of which is frequent in BC and is associated with poor prognosis [28]. The PTEN encoding mRNA contains a putative miR-181a binding site within its 3′-UTR (Additional file 1: Fig. S2). Since PTEN has been previously identified as a direct target of miR-181a and is a key upstream inhibitor of Akt–mTOR signaling [29], we conjectured that the enhancing effects of TEMo-secreted miR-181a on AKT activation may be attributed to modulating PTEN. To this end, we transfected MDA-MB-231 BC cells with miR-181a mimic and indicated that miR-181a overexpression led to a significant reduction of PTEN expression at both mRNA and protein levels (Fig. 8F), showing that miR-181a may regulate PTEN in BC cells. To highlight the functional paracrine effects of monocytes, we first measured the expression level of PTEN in MDA-MB-231 cells that were incubated with 100 μg/mL exosomes derived from CAF- or NF-educated monocytes. RT-qPCR results showed that incubation of MDA-MB-231 cells with TEMo-Exo led to a significantly lower level of PTEN expression than those incubated with exosomes derived from control monocytes or NEMo (Fig. 8G). Next, to reveal the functional effect of TEMo-secreted miR-181a on PTEN expression, MDA-MB-231 cells were transfected with miR-181a inhibitor (100 nM) and stimulated with 100 μg/mL CAF-educated monocyte exosomes for 24 h. Importantly, following transfection with the miR-181a inhibitor, the inhibitory effect of CAF-educated monocyte exosomes on the PTEN mRNA expression level was partially rescued in MDA-MB-231 cells (Fig. 8G), suggesting that down-regulation of PTEN is specific and largely dependent on the transfer of this exosome-shuttled miRNA. Next to MDA-MB-231 cells, we also examined the effect of exosomal transfer of miR-181a on another BC-derived cell line, MCF-7. The qRT-PCR results revealed that incubating α-amanitin-treated MCF-7 with 100 μg/mL TEMo-Exo resulted in a gradual increase in the level of miR-181a at different time points, whereas there was no significant difference in the level of this miRNA in control cell groups. Moreover, results showed that the exosomal transfer of miR-181a from TEMo reduced PTEN expression in MCF-7 cells (Additional file 1: Fig. S3). Altogether, these findings support the notion that exosomes derived from TEMo play a role as an activator whereby they activate AKT signaling. We also came up with a plausible model in which TEMo-secreted miR-181a activates AKT signaling through regulating PTEN in BC cells.

## Discussion

In recent years, the TME has attracted increasing attention due to its critical roles in multiple stages of disease progression, particularly tumor immunosuppression, local resistance, distant metastasis, and targeted therapy outcome [30–32]. Although BC is considered a heterogeneous disease characterized by aberrant mutations in mammary tumor cells, it is now clearly apparent that such tumors are also diverse by the nature of their microenvironmental composition and the activity of their stromal cell proportions [33, 34]. CAFs, as one of the most important stromal components in the TME, confer a mesenchymal-like phenotype to malignant epithelial cells and support tumor growth and metastasis [35]. In addition to playing tumor-promoting roles in the initiation and progression of tumor growth, CAFs were also shown to sculpt the TME [36]. It was proposed that the tumor-promoting secretome of CAFs may exert potent remodeling effects on tumor immunity, affecting innate immune cell recruitment and activation and polarizing the adaptive immune response [37].

There exists a close relationship between TAMs and CAFs, as TAMs are the most common type of immune cell in close proximity to CAF-populated areas [3]. High infiltration of TAMs in tumors correlates with tumor aggressiveness and reduces overall and recurrence-free survival [38, 39]. There are several TME-derived factors that trigger monocyte recruitment into tumor tissues by a hypoxia-induced chemoattractant gradient [40, 41]. Previous studies demonstrated that monocyte chemotactic protein-1 (MCP-1) and stromal cell-derived factor-1 (SDF-1) take part in monocyte recruitment into breast tumors as chemotactic cytokines secreted by stromal cells [42, 43]. In this study, we demonstrated that CAFs obtained from invasive BC could recruit and subsequently differentiate monocytes into M2-like pro-tumoral macrophages in terms of both phenotypic features and functions, in contrast to fibroblasts obtained from normal breast (Figs. 1, 2). Consistently, there are several studies indicating that CAFs induce the M2 polarization of TAMs, which is characterized by an IL-12^low^ IL-10^high^ phenotype and up-regulating M2-specific markers CD163 and CD206 [4, 9, 43, 44]. Mounting evidence suggests that redox signaling plays a role in macrophage polarization [45]. It is thought that ROS in macrophages is required for the phagocytosis and clearance of apoptotic cells. However, sustaining a high amount of ROS may not be tolerated by macrophages because inducible ROS has been shown to trigger macrophage apoptosis [45, 46]. The involvement of ROS in regulating the functional reprogramming of macrophages may determine the macrophage’s ability to mediate phagocytosis [47]. Previous studies have shown that increasing the levels of ROS in the TME can contribute to the differentiation of M2-polarized macrophages [48, 49]. Consistently, we found that the M2 phenotype transformation induced by CAF was concomitant with increased ROS production in differentiated THP-1 macrophages. Despite ROS levels being reduced during M1/M2 macrophage polarization, CAF-induced M2-like macrophages still appear to produce a higher level of ROS than THP-1 control monocytes (Fig. 2D, E). These observations implicated ROS as being a component in the M2 phenotype whose levels may be adjusted by tumor stromal cells.

Though the CAF secretome is still not fully characterized, there is evidence that CAF-secreted cytokines and growth factors may trigger the immunosuppressive functions that involve various immune cells and stages of anti-tumoral activity [50, 51]. Recent studies have highlighted the direct implication of CAFs in the tumor immunosuppressive microenvironment by excluding T-cells from tumors [52]. In line with these findings, our data revealed that CAF-educated monocytes were able to considerably suppress T-cell proliferation, in contrast to their normal counterparts. Moreover, treatment of T-cells with exosomes derived from TEMo or NEMo exhibited effects comparable to co-culture with educated monocytes, indicating the significance of the biological functions of exosomes (Fig. 4). Therefore, by educating monocytes into a distinct population of macrophages that exhibit an M2-like phenotype, CAFs may exert their immunosuppressive effects through driving T-cell exclusion in an indirect fashion.

Apart from reciprocal communication with each other, both CAFs and TAMs are in a dynamic interaction with the tumor cells in the tumor *milieu* [4]. In BC, TAMs comprise about half of the cell tumor mass and can in turn facilitate tumor growth and metastasis [53]. Supporting this notion are the observations whereby the cross-talk between M2-polarized TAMs and tumor cells is responsible for inducing EMT to promote tumor metastasis [54–56]. Previous studies have shown that immunosuppressive cytokines and survival factors secreted by TAMs promote BC progression and metastasis [57]. Since TAMs may secrete paracrine factors that drive the phenotypic and signaling pathway alterations across the tumor cohort [58], we here sought to investigate whether exosomes secreted from TEMo affect tumor growth. We revealed that TEMo-Exo augmented BC cell proliferation and migration as well as the expression of EMT markers, while exosomes derived from control-educated monocytes had no effect on the aggressive behavior of BC cells (Fig. 5). Importantly, TEMo-Exo, but not NEMo-Exo, exhibited the potential to induce tumor growth and enhanced the expression of the tumor proliferation marker Ki-67 in BC xenograft tumors (Fig. 6). Tumor cell-bearing athymic nude mice lack a thymus to produce T-cells but possess B-cells capable of producing antibodies in a T-cell-independent way. This model contains intact innate immunity with enhanced natural killer (NK) cell activity, which can reduce the rate of engraftment, growth, and metastasis formation. The subcutaneous heterotopic model is the most common one due to its relative simplicity in design and evaluation, provides realistic heterogeneity of tumor cells, and allows for rapid analysis of the human tumor response to a treatment regimen [59]. As the activity of NK cells tends to increase with age, we utilized younger mice (6 weeks old) to enhance the engraftment rate, and, thus, the reproducibility of the assays. Our findings led us to suggest that CAFs may aid tumor growth in an athymic nude mouse model of BC by educating monocytes and their derivative exosomes.

Although CAFs are important in the formation of TME and in interacting with tumor cells, the effects of their secretome on the behavior of immune stromal cells, particularly in terms of tumor progression, require further investigation. Most current studies focus on the cytokines or regulatory protein factors that are secreted by macrophages. However, close attention needs to be paid to miRNAs, which are considered key regulators of tumorigenesis and, more importantly, are selectively secreted by TAM-derived exosomes. Because aberrant expression and function of miRNAs are common characteristics of malignant cells, these small RNAs provide important opportunities for the development of future miRNA-based therapies for human cancers such as BC [26, 60]. miR-181a was found to be a miRNA associated with BC progression [61, 62], and up-regulation of which was detected in plasma samples of breast ductal carcinoma patients. Additionally, our data demonstrated that there are differences between exosomes derived from TEMo and their normal counterparts in terms of both miR-181a content and BC cell progression (Fig. 7). To define a possible mechanism through which miR-181a may promote BC cell progression, we explored the downstream mechanism of miR-181a and its relation with the relevant intracellular signaling. Studies on the downstream targets of miR-181a have revealed PTEN as a key tumor-suppressor gene. PTEN constitutes a main inhibitory node in Akt signaling as it functions as a PIP3 phosphatase [63]. Loss of PTEN function results in constitutive activation of AKT which plays a crucial role in breast tumorigenesis [64]. Herein, our functional analyses validated the oncogenic role of miR-181a by regulating PTEN to promote the more aggressive phenotypes of BC cells by activating AKT signaling. As miR-181a overexpression recapitulated the effects of TEMo-Exo on BC cells, it is proposed that exosomal transfer of miR-181a contributes to promoting BC cell progression (Figs. 7, 8).

## Conclusions

**Fig. 9:**
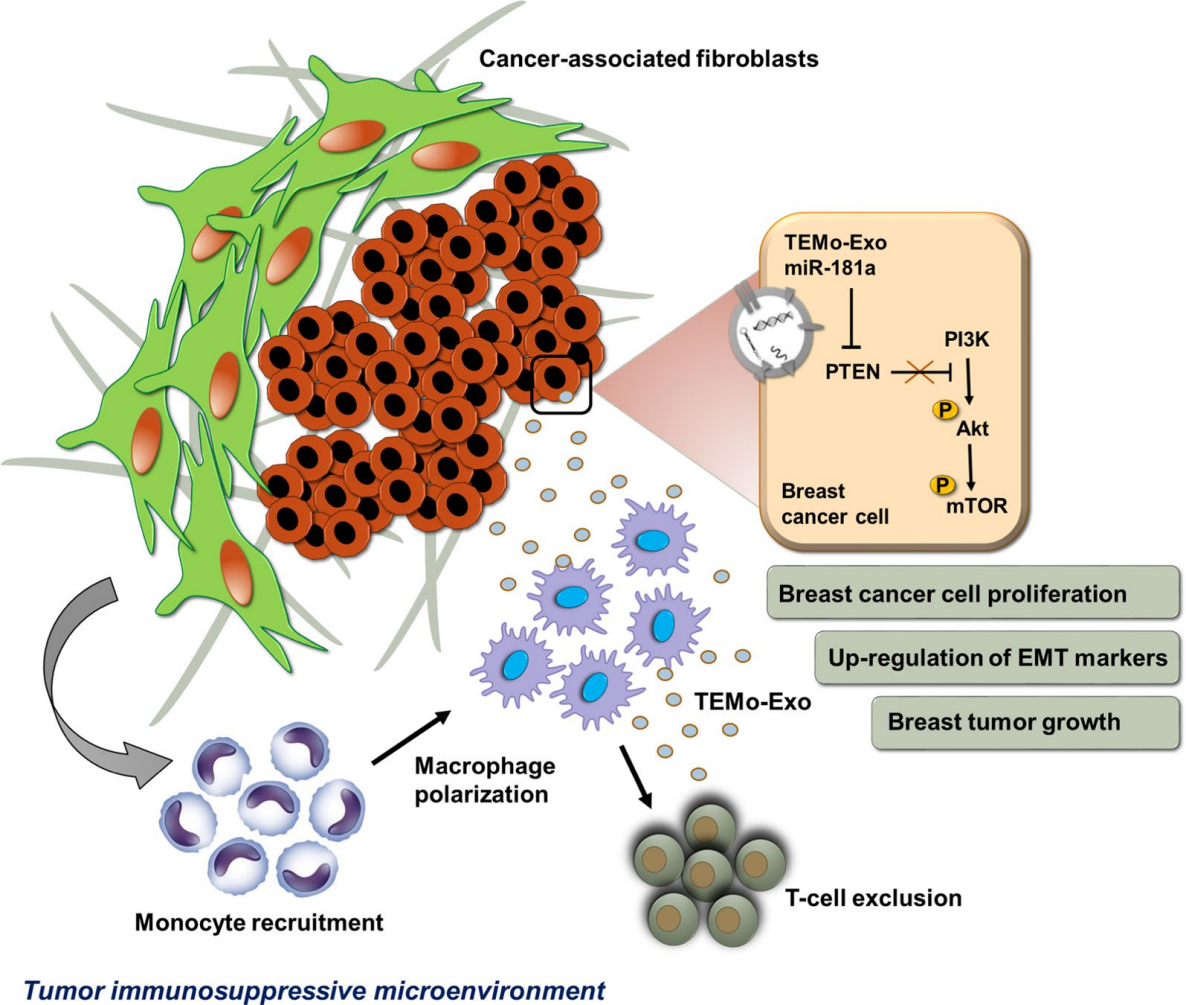
A proposed model illustrating how CAFs drive a more aggressive phenotype in BC by recruiting monocytes in a paracrine manner. CAFs contribute to preparing an immunosuppressive TME by recruiting monocytes and educating them into a distinct population of macrophages exhibiting an M2-like phenotype. As a plausible mechanism, exosomal transfer of miR-181a from CAF-educated monocytes is partly associated with activating Akt signaling and up-regulating EMT markers, thereby promoting breast tumor progression and growth

In conclusion, this study proposed a model illustrating how CAF secretome induces a tumor immunosuppressive microenvironment by recruiting monocytes. Our study provided the first evidence that exosomal transfer of a miRNA from CAF-educated monocytes may promote BC cell progression through activating Akt signaling (Fig. 9). Given that a miRNA has many targets and may function via different pathways, the current findings do not exclusively demonstrate the exact mechanism by which tumor-educated monocytes affect BC progression. However, our findings suggest that breast CAFs play a crucial role in shaping an immunosuppressive TME by recruiting monocytes in a paracrine manner. We proposed that exosomal miR-181a shuttled by these CAF-activated monocytes was in part associated with activating Akt signaling, thereby promoting BC progression. Obviously, uncovering the pro-tumoral function of CAFs through educating monocytes into a distinct population of macrophages exhibiting an M2-like phenotype holds great promise in targeted cancer therapy. In this regard, a deeper understanding of the intercellular miRNA communication between immune stromal and tumor cells may provide potential targets for therapeutic intervention against BC.

## Supplementary Information

**Additional file 1: Table S1.** The clinicopathological characteristics of patients with invasive breast carcinoma. **Figure S1. **Western blot analysis showed the expression of CAF-specific markers (α-SMA and FAP) and CAF-derived cytokines (IL-6, and TGF-β) at passages (P) 2 and 5, confirming CAF activation status throughout the experiments. **Figure S2.** Functional enrichment analysis. **Figure S3.** Exosomal transfer of miR-181a from TEMo inhibits PTEN expression in MCF-7 BC cells.

## Data Availability

The data supporting the findings of this study are available from the corresponding author upon reasonable request.

## Abbreviations

BC: Breast cancer
TME: Tumor microenvironment
CAFs: Cancer-associated fibroblasts
NFs: Normal fibroblasts
TAMs: Tumor-associated macrophages
TEMo: Tumoral fibroblast-educated monocytes
NEMo: Normal fibroblasts-educated monocytes
PBMCs: Peripheral blood mononuclear cells
EMT: Epithelial-to-mesenchymal transition
CM: Conditioned media
SMA: Smooth muscle actin
FAP: Fibroblast-activation protein
PD-1: Programmed cell death protein 1
MHC: Major histocompatibility complex
MCP-1: Monocyte chemotactic protein-1
SDF-1: Stromal cell-derived factor-1
Exo: Exosomes
MiRNA or miR: MicroRNA
DIANA: DNA Intelligent Analysis
KEGG: Kyoto Encyclopedia of Genes and Genomes
PMA: Phorbol 12-myristate 13-acetate
H&E: Hematoxylin and eosin
IHC: Immunohistochemistry
ELISA: Enzyme-linked immunosorbent assay
TEM: Transmission electron microscopy
DLS: Dynamic light scattering
CFSE: Carboxyfluorescein succinimidyl ester
PI: Propidium iodide
ROS: Reactive oxygen species
