# Supplementary material for: Monocytes educated by cancer-associated fibroblasts secrete exosomal miR-181a to activate AKT signaling in breast cancer cells
Source: J Transl Med. 2022 Dec 3;20:559. doi: 10.1186/s12967-022-03780-2 (PMC9719191; doi:10.1186/s12967-022-03780-2)
Supplement: Supplementary file 1 — Additional file 1: Table S1. The clinicopathological characteristics of patients with invasive breast carcinoma. Figure S1. Western blot analysis showed the expression of CAF-specific markers (α-SMA and FAP) and CAF-derived cytokines (IL-6, and TGF-β) at passages (P) 2 and 5, confirming CAF activation status throughout the experiments. Figure S2. Functional enrichment analysis. Figure S3. Exosomal transfer of miR-181a from TEMo inhibits PTEN expression in MCF-7 BC cells. [file 12967_2022_3780_MOESM1_ESM.pptx]

## Slide 1
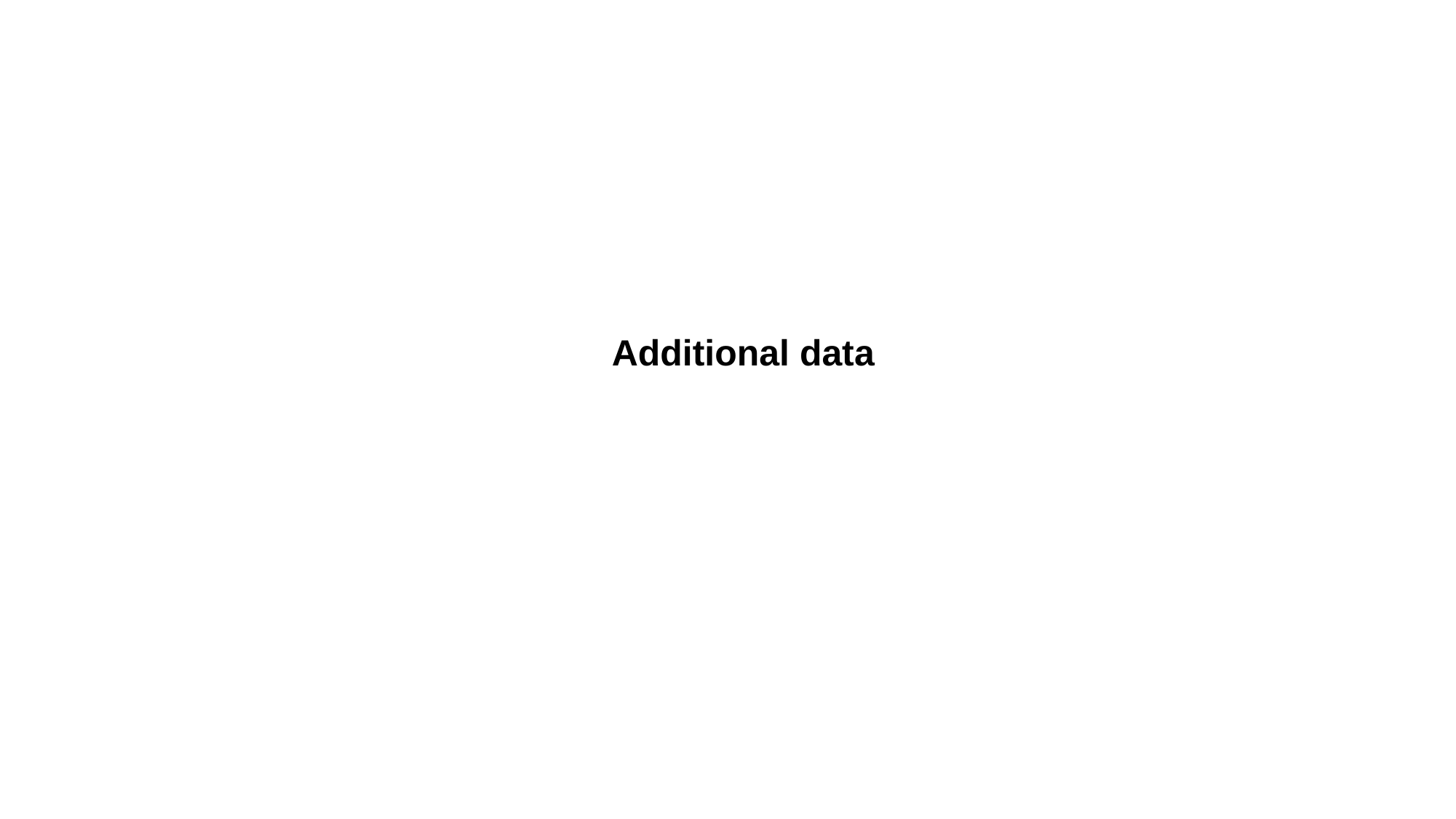

Additional data

## Slide 2
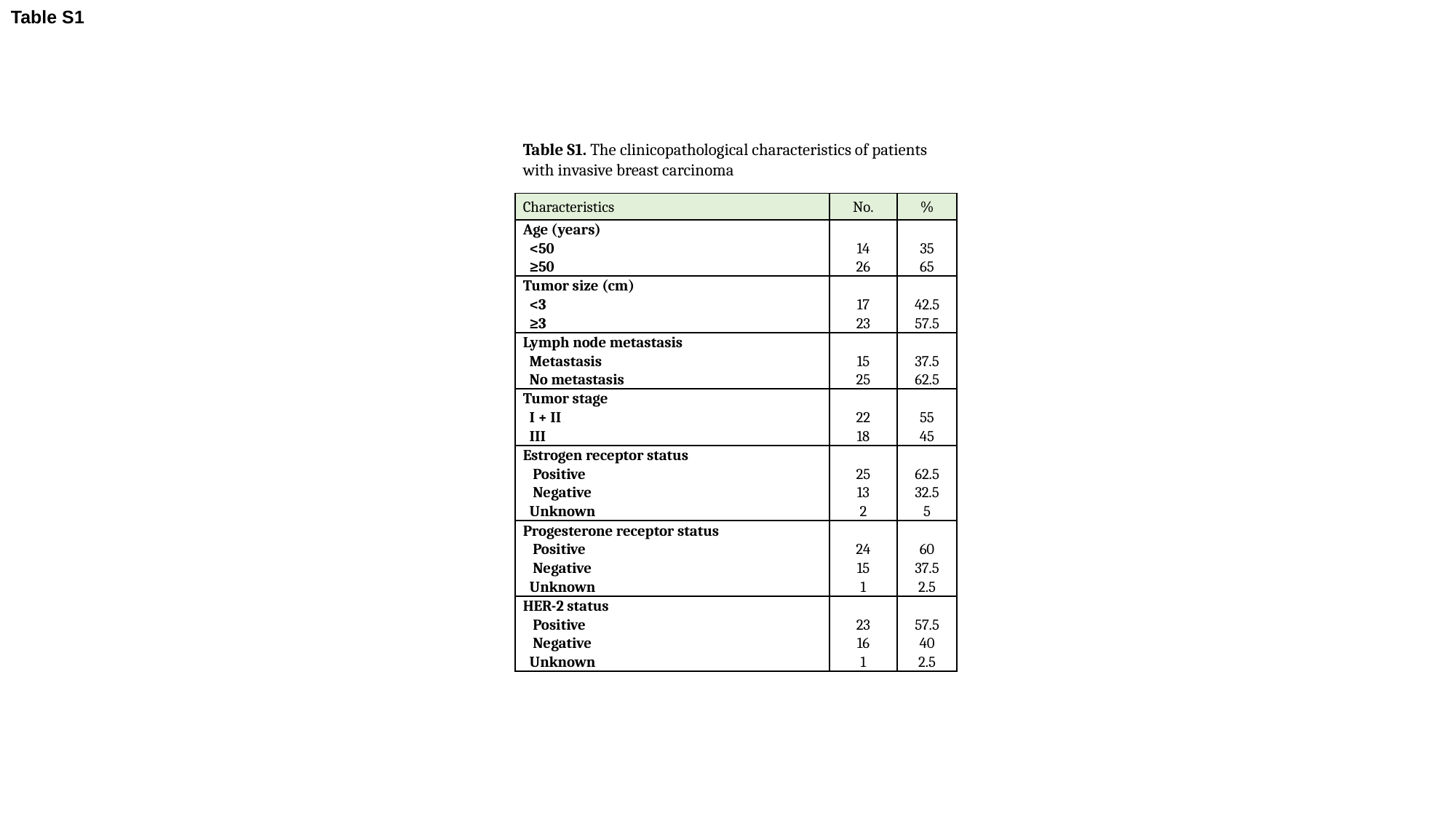

Table S1
| Table S1. The clinicopathological characteristics of patients with invasive breast carcinoma | | |
| --- | --- | --- |
| Characteristics | No. | % |
| Age (years) <50 ≥50 | 14 26 | 35 65 |
| Tumor size (cm) <3 ≥3 | 17 23 | 42.5 57.5 |
| Lymph node metastasis Metastasis No metastasis | 15 25 | 37.5 62.5 |
| Tumor stage I + II III | 22 18 | 55 45 |
| Estrogen receptor status Positive Negative Unknown | 25 13 2 | 62.5 32.5 5 |
| Progesterone receptor status Positive Negative Unknown | 24 15 1 | 60 37.5 2.5 |
| HER-2 status Positive Negative Unknown | 23 16 1 | 57.5 40 2.5 |

## Slide 3
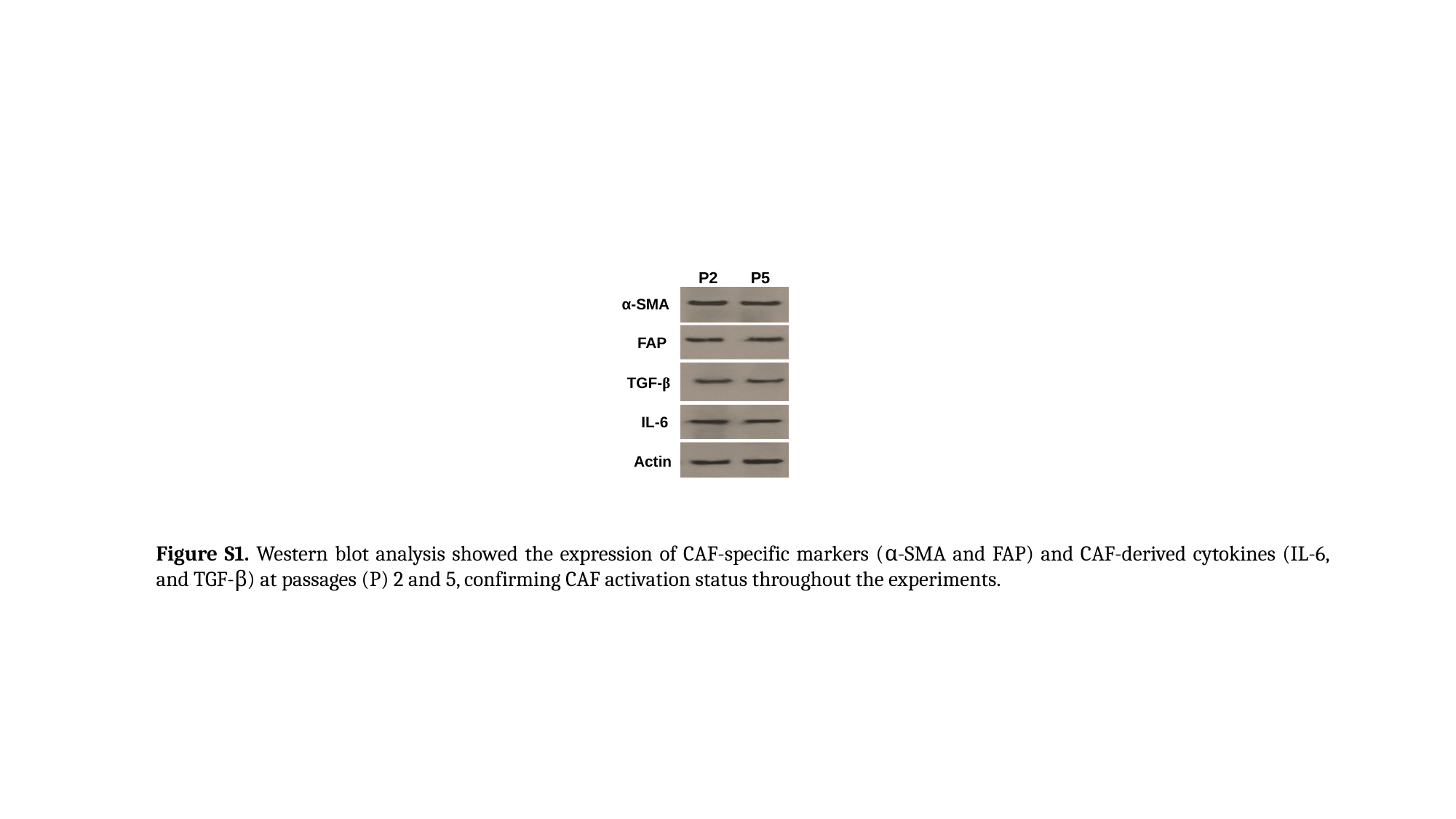

P2
P5
α-SMA
FAP
TGF-β
IL-6
Actin
Figure S1. Western blot analysis showed the expression of CAF-specific markers (α-SMA and FAP) and CAF-derived cytokines (IL-6, and TGF-β) at passages (P) 2 and 5, confirming CAF activation status throughout the experiments.

## Slide 4
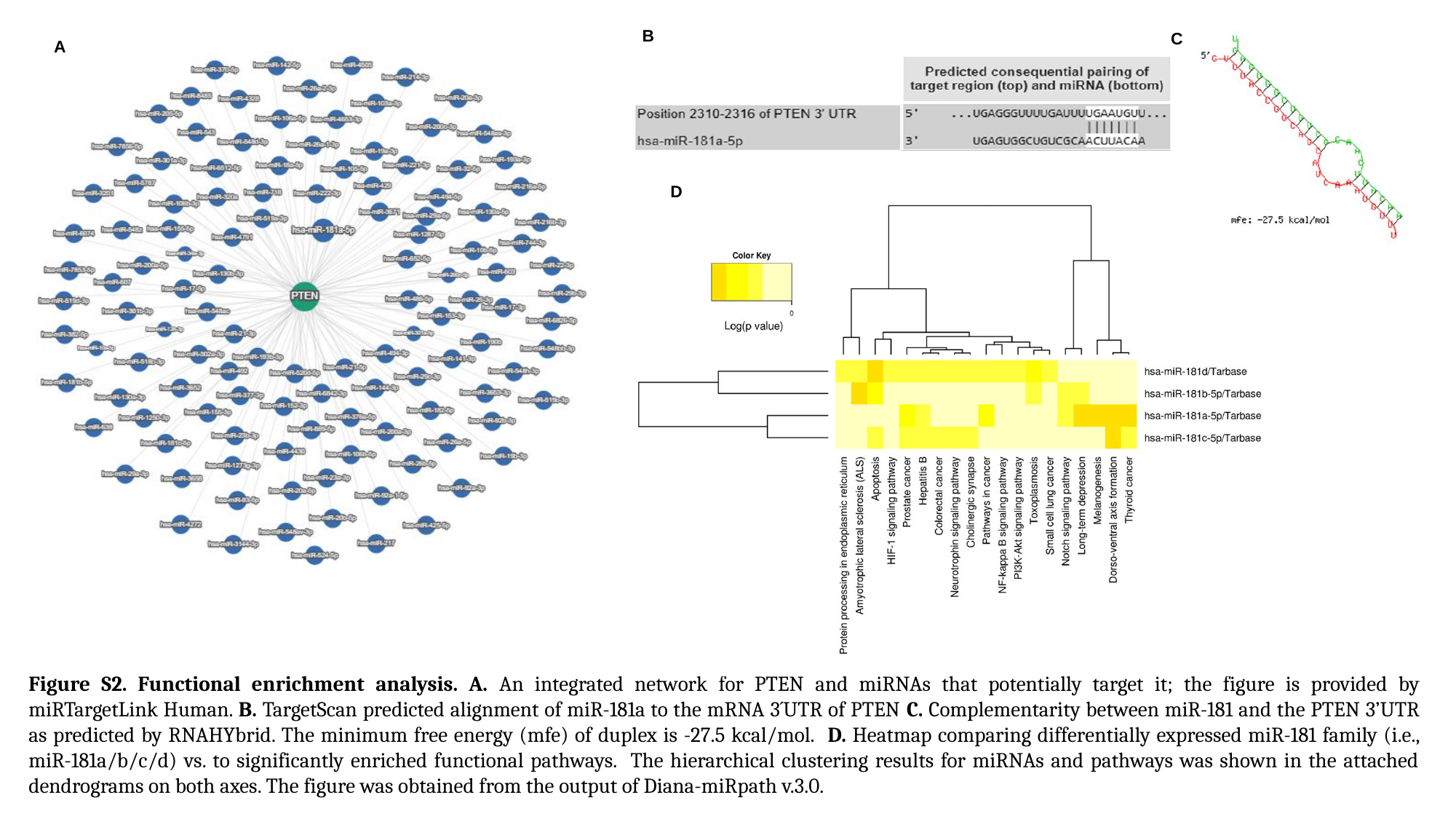

B
C
A
D
Figure S2. Functional enrichment analysis. A. An integrated network for PTEN and miRNAs that potentially target it; the figure is provided by miRTargetLink Human. B. TargetScan predicted alignment of miR-181a to the mRNA 3´UTR of PTEN C. Complementarity between miR-181 and the PTEN 3’UTR as predicted by RNAHYbrid. The minimum free energy (mfe) of duplex is -27.5 kcal/mol. D. Heatmap comparing differentially expressed miR-181 family (i.e., miR-181a/b/c/d) vs. to significantly enriched functional pathways. The hierarchical clustering results for miRNAs and pathways was shown in the attached dendrograms on both axes. The figure was obtained from the output of Diana-miRpath v.3.0.

## Slide 5
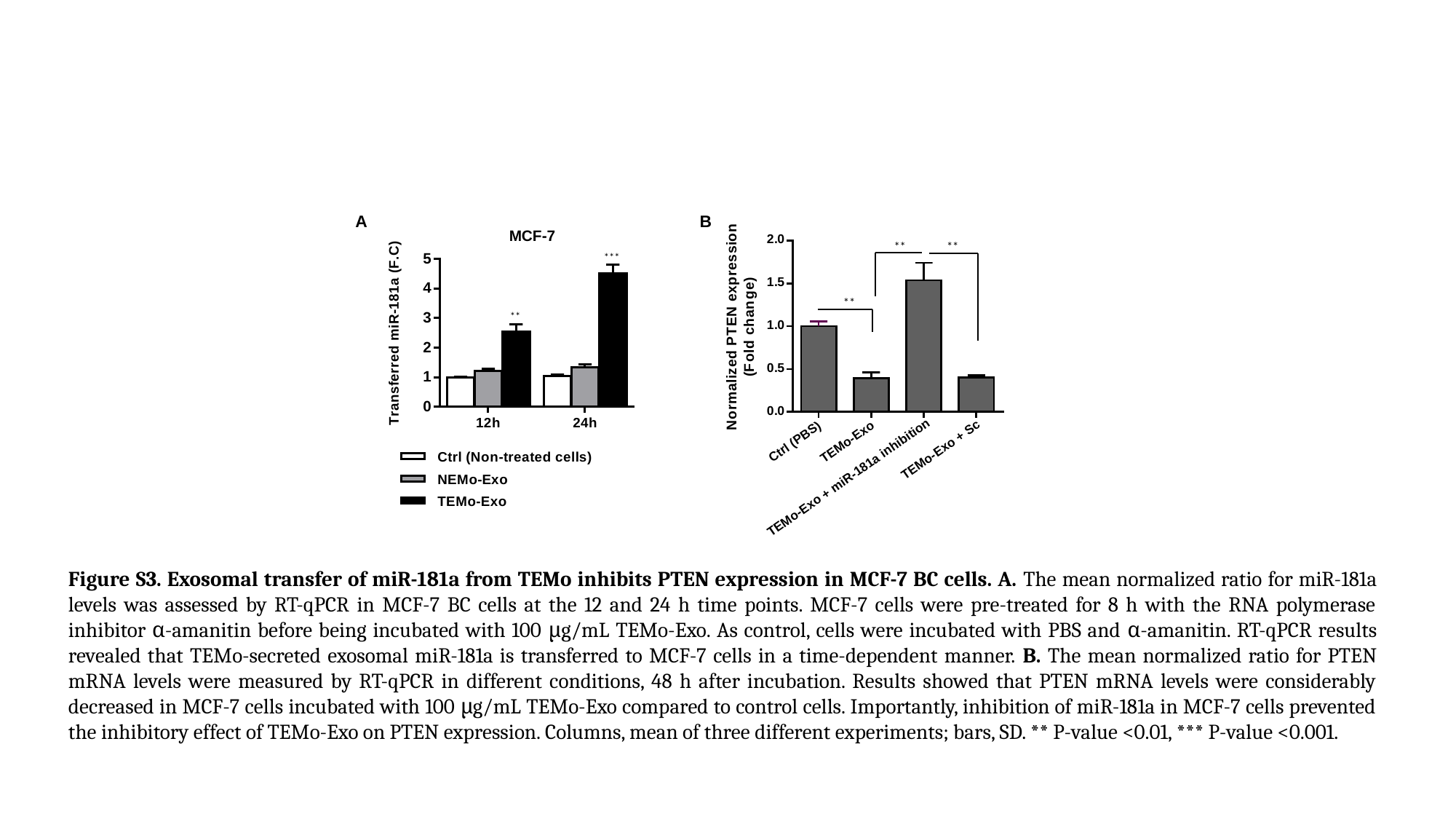

B
**
**
**
A
MCF-7
***
**
Figure S3. Exosomal transfer of miR-181a from TEMo inhibits PTEN expression in MCF-7 BC cells. A. The mean normalized ratio for miR-181a levels was assessed by RT-qPCR in MCF-7 BC cells at the 12 and 24 h time points. MCF-7 cells were pre-treated for 8 h with the RNA polymerase inhibitor α-amanitin before being incubated with 100 μg/mL TEMo-Exo. As control, cells were incubated with PBS and α-amanitin. RT-qPCR results revealed that TEMo-secreted exosomal miR-181a is transferred to MCF-7 cells in a time-dependent manner. B. The mean normalized ratio for PTEN mRNA levels were measured by RT-qPCR in different conditions, 48 h after incubation. Results showed that PTEN mRNA levels were considerably decreased in MCF-7 cells incubated with 100 μg/mL TEMo-Exo compared to control cells. Importantly, inhibition of miR-181a in MCF-7 cells prevented the inhibitory effect of TEMo-Exo on PTEN expression. Columns, mean of three different experiments; bars, SD. ** P-value <0.01, *** P-value <0.001.
